# Apoptosis Signal-Regulating Kinase 1 Mediates MPTP Toxicity and Regulates Glial Activation

**DOI:** 10.1371/journal.pone.0029935

**Published:** 2012-01-10

**Authors:** Kang-Woo Lee, Xin Zhao, Joo-Young Im, Hilary Grosso, Won Hee Jang, Teresa W. Chan, Patricia K. Sonsalla, Dwight C. German, Hidenori Ichijo, Eunsung Junn, M. Maral Mouradian

**Affiliations:** 1 Center for Neurodegenerative and Neuroimmunologic Diseases, Department of Neurology, UMDNJ-Robert Wood Johnson Medical School, Piscataway, New Jersey, United States of America; 2 Department of Biochemistry, College of Medicine, Inje University, Busan, Korea; 3 Department of Psychiatry, University of Texas Southwestern Medical Center, Dallas, Texas, United States of America; 4 Laboratory of Cell Signaling, Graduate School of Pharmaceutical Sciences, The University of Tokyo, Tokyo, Japan; National Institutes of Health, United States of America

## Abstract

Apoptosis signal-regulating kinase 1 (ASK1), a member of the mitogen-activated protein kinase 3 family, is activated by oxidative stress. The death-signaling pathway mediated by ASK1 is inhibited by DJ-1, which is linked to recessively inherited Parkinson's disease (PD). Considering that DJ-1 deficiency exacerbates the toxicity of the mitochondrial complex I inhibitor 1-methyl-4-phenyl-1,2,3,6-tetrahydropyridine (MPTP), we sought to investigate the direct role and mechanism of ASK1 in MPTP-induced dopamine neuron toxicity. In the present study, we found that MPTP administration to wild-type mice activates ASK1 in the midbrain. In ASK1 null mice, MPTP-induced motor impairment was less profound, and striatal dopamine content and nigral dopamine neuron counts were relatively preserved compared to wild-type littermates. Further, microglia and astrocyte activation seen in wild-type mice challenged with MPTP was markedly attenuated in ASK1^−/−^ mice. These data suggest that ASK1 is a key player in MPTP-induced glial activation linking oxidative stress with neuroinflammation, two well recognized pathogenetic factors in PD. These findings demonstrate that ASK1 is an important effector of MPTP-induced toxicity and suggest that inhibiting this kinase is a plausible therapeutic strategy for protecting dopamine neurons in PD.

## Introduction

Parkinson's disease (PD) is a common neurodegenerative disorder in which dopaminergic neurons of the substantia nigra bear the brunt of the pathology. A consistent biochemical abnormality in this brain region documented in postmortem studies is oxidative stress with distinct markers of damage to cellular proteins, lipids and DNA [1]. These findings, along with the realization that agents that are toxic to dopaminergic neurons *in vitro* and *in vivo* cause increased intracellular levels of reactive oxygen species, have led to the use of those toxins to generate animal models of PD. Among these toxins, MPTP is a mitochondrial complex I inhibitor that causes parkinsonian features in humans and primates, and recapitulates dopaminergic deficits in the nigrostriatal pathway of mice [2], [3]. Therefore, MPTP is widely used as a tool to study the molecular events that lead to degeneration of dopaminergic neurons in animal models of PD and to test potentially neuroprotective agents.

Several genes have been linked to familial forms of PD. Among these, DJ-1, which causes recessively inherited disease when mutated, encodes a neuroprotective protein that has antioxidant activity [4]. In addition, we reported previously that DJ-1 achieves its cytoprotective function by interfering with the activation of Apoptosis Signal-Regulating Kinase 1 (ASK1) signaling pathway [5], suggesting that the latter might be involved in the pathogenesis of PD.

ASK1 is a member of the MAP3 kinase family that activates JNK and p38 kinase pathways [6], [7]. In addition to Fas-induced, Daxx-mediated activation [8], ASK1 is activated by various stimuli including oxidative stress [9], [10], endoplasmic reticulum stress [11] and TNF-α [12], and relays those signals to JNK and p38 [8] leading to apoptosis [9]. A number of studies have suggested that ASK1 is functional in neural tissues. Its mRNA is expressed in brain, including in cortex, hippocampus, olfactory bulb and striatum [13], and expression of a constitutively active form of ASK1 induces neurite outgrowth in the rat pheochromocytoma PC12 cell line [14]. In addition, the requirement of ASK1 activation has been demonstrated in an ALS mouse model expressing mutant form of SOD1 [15]. In a recent postmortem study of PD affected brains, ASK1 was found to be activated in substantia nigra neurons compared to control brains [16]. Further, ASK1^−/−^ primary neurons are resistant to cell death induced by ER stress, proteasome dysfunction, and expanded polyglutamine expression [11], and ASK1 knockdown using shRNA in the mouse nigra diminishes 6-hydroxydopamine toxicity [16]. Thus, ASK1 is a key player in mediating the effects of various cellular insults.

Here, we show that MPTP administration activates ASK1 in the substantia nigra of wild-type mice, while deleting the ASK1 gene confers relative resistance to this toxin demonstrated by biochemical, histopathological and behavioral analyses. Our results indicate that blocking ASK1 expression can mitigate the dopaminergic neuron loss that follows MPTP intoxication and suppresses glial activation in the nigra and striatum. These findings suggest that ASK1 signaling plays an important role in MPTP-induced toxicity in mice by linking the oxidative stress generated by MPTP and the associated neuroinflammation. Controlling ASK1 activity may ultimately help design new neuroprotective therapeutic interventions for PD and other neurodegenerative disorders in which oxidative stress plays a significant pathogenetic role.

## Results

### ASK1 is activated by oxidative stress in cultured cells and *in vivo*

**Figure 1:**
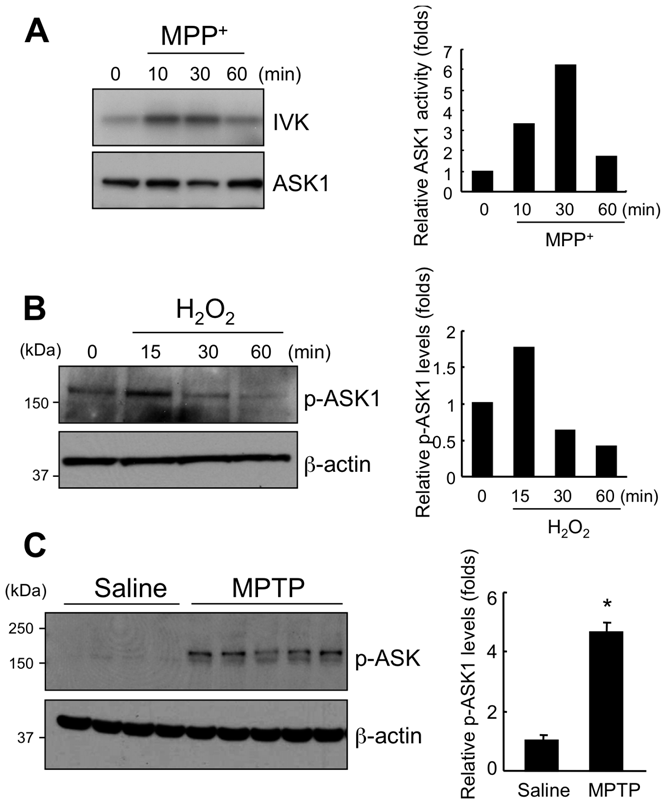
Oxidative stress-induced ASK1 activation. (A) ASK1 activation by MPP^+^. SH-SY5Y cells were challenged with 5 mM MPP^+^ and ASK1 activity measured by in vitro kinase (IVK) assay. Total ASK1 expression was checked by Western blotting. The bar graph on the right shows quantification of band intensities. (B) ASK1 phosphorylation with hydrogen peroxide treatment. Rat primary cortical neurons were challenged with 1 mM H_2_O_2_ followed by Western blotting for phospho-ASK1. The bar graph on the right shows quantification of the Western blot data. (C) ASK1 phosphorylation in MPTP-injected mice. Wild-type mice were injected with MPTP (20 mg/kg every 2 h×4) or saline intraperitoneally and sacrificed 90 minutes after the last injection. Midbrains containing substantia nigra from four saline treated mice and 5 MPTP challenged mice were homogenized and lysed for Western blotting with phospho-ASK1 antibody and total ASK1 antibody. The bar graph shows quantification of the Western blot data. * p<0.05.

Since ASK1 is known to be a key player in oxidative stress-induced cell signaling, we examined whether MPTP-induced dopaminergic cell death is associated with ASK1 activation. First, dopaminergic neuroblastoma SH-SY5Y cells were challenged with MPP^+^, and processed for *in vitro* kinase assay of ASK1. A significant increase in ASK1 activity was seen as early as 10 minutes after MPP^+^ treatment (Fig. 1A). We also observed ASK1 activation in primary rat cortical neurons following H_2_O_2_ challenge (1 mM) detected by a transient increase in the amount of phosphorylated ASK1 (Fig. 1B) as reported previously [17]. Next, we investigated the levels of phosphorylated ASK1 following MPTP administration into mice. Wild-type C57BL/6J mice were injected with MPTP (20 mg/kg every 2 h×4) or saline intraperitoneally and sacrificed 90 min after the last injection. Ventral midbrains containing substantia nigra pars compacta were processed for Western blotting using anti-phophorylated ASK1 antibody. MPTP administration significantly increased phosphorylated ASK1 level compared to saline-treated animals (Fig. 1C) consistent with previous reports [18], [19]. These findings indicate that ASK1 is activated *in vitro* and *in vivo* after insults that produce oxidative stress.

### ASK1 null mice are relatively resistant to MPTP

**Figure 2:**
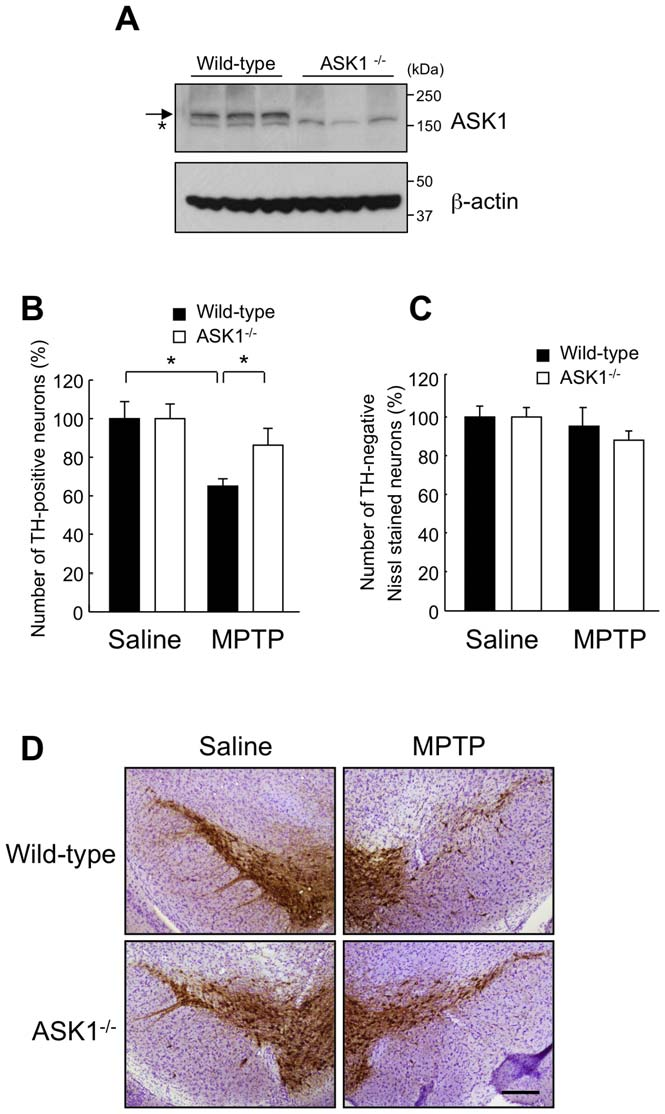
Nigral dopaminergic neurons are protected against MPTP in ASK1^−/−^ mice. (A) Western blot for ASK1 in substantia nigra tissue lysates from wild-type and ASK1^−/−^ mice shows loss of the specific band in the latter group (arrow). The asterisk marks a nonspecific band. (B) Stereological counting of TH positive nigral dopamine neurons following MPTP or saline administration. * p<0.05. (C) Number of Nissl stained TH negative neurons. Saline-WT (n = 5), Saline-ASK1^−/−^ (n = 5), MPTP-WT (n = 4), MPTP-ASK1^−/−^ (n = 5). (D) Representative images of TH immunohistochemistry of midbrain sections from wild-type and ASK1^−/−^ mice treated with saline or MPTP. Scale bar = 200 µm.

To address the role of ASK1 in the pathogenesis of PD, we compared the susceptibility of wild-type and ASK1^−/−^ mice to MPTP. First, loss of ASK1 expression was confirmed by Western blotting of substantia nigra tissue lysates from ASK1^−/−^ mice (Fig. 2A). MPTP was administered over 5 days (30 mg/kg/day) and animals were sacrificed 14 days after the last injection. The integrity of nigral dopaminergic neurons was assessed by stereological counting of TH positive neurons. Compared to about 35% loss of TH-positive neurons due to MPTP in wild-type mice, this figure was only about 13% in ASK1^−/−^ animals (Fig. 2B). On the other hand, stereological counting of TH-negative, Nissl-stained neurons showed no change after MPTP in either group (Fig. *2*C). The finding that TH-positive neurons decreased while TH-negative/Nissl stained neurons did not increase after MPTP compared to saline indicates degeneration of TH-positive neurons rather than merely decreased TH expression. Representative immunohistochemical images show marked degeneration of nigral dopaminergic neurons in MPTP challenged wild-type mice but a significantly attenuated effect in ASK1^−/−^ mice (Fig. 2D).

**Figure 3:**
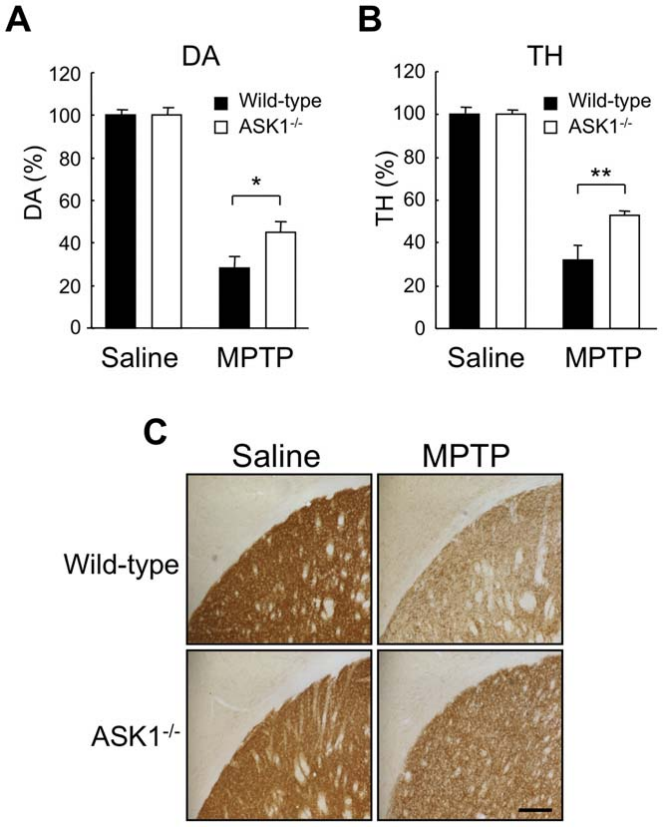
Indices of dopamine nerve terminal integrity in the striatum show protection against MPTP in ASK1^−/−^ mice. (A) Striatal dopamine content following MPTP or saline administration. * p<0.05. (B) TH content measured by ELISA in striatal tissue. ** p<0.01. Saline-WT (n = 5), Saline-ASK1^−/−^ (n = 5), MPTP-WT (n = 4), MPTP-ASK1^−/−^ (n = 5). (C) Representative images of immunohistochemical images for TH in striatum. Scale bar = 200 µm.

Striatal dopamine depletion is an important measure of dopaminergic neuron degeneration in PD and MPTP models. Two weeks after MPTP administration, striatal dopamine content decreased down to 28% in wild-type mice compared to saline injected animals. On the other hand, dopamine content in ASK1^−/−^ mice decreased down to 45% (p<0.05) (Fig. 3A). Striatal tyrosine hydroxylase content measured by ELISA also showed a smaller decline after MPTP exposure in ASK1^−/−^ mice compared to wild-type animals (32% residual TH in wild-type mice compared to 53% in ASK1^−/−^ mice; p<0.01) (Fig. 3B). Immunohistochemical staining of the striatum for TH revealed a profile consistent with the ELISA data indicating that striatal dopaminergic terminals of ASK1^−/−^ mice were relatively spared compared to their wild-type counterparts (Fig. *3*C).

To exert its toxicity *in vivo*, MPTP is first converted to MPP^+^ by MAO-B activity. To confirm that the attenuated response of ASK1^−/−^ mice to MPTP is not due to altered MPTP metabolism in this mouse line, we measured striatal MPP^+^ levels after MPTP intoxication. Ninety minutes after a single IP injection of 30 mg/kg MPTP, striatal MPP^+^ levels measured by HPLC were 3.65±0.42 ng/mg protein (n = 8) for ASK1^−/−^ mice compared with 3.88±0.55 ng/mg protein for wild-type mice (n = 6). This lack of a difference indicates that MPTP metabolism is not altered as a result of ASK1 deletion, and, therefore, this possibility cannot account for the attenuated response of ASK1^−/−^ mice to MPTP toxicity.

**Figure 4:**
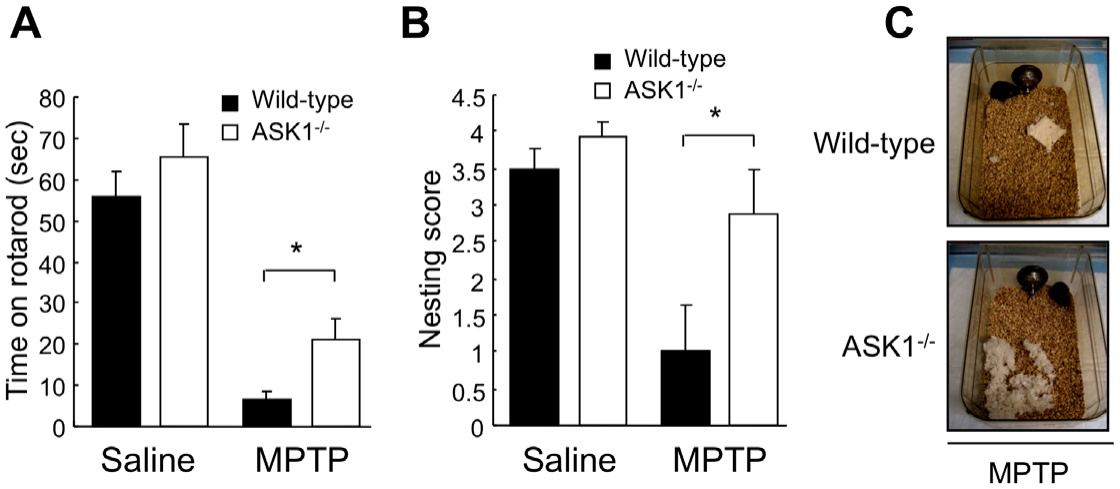
Preserved behavioral responses in MPTP-treated ASK1^−/−^ mice compared to MPTP-treated wild-type mice. (A) Performance on the Rotarod test one day post-MPTP. Saline-WT (n = 3), Saline-ASK1^−/−^ (n = 6), MPTP-WT (n = 3), MPTP-ASK1^−/−^ (n = 4). * p<0.05. (B, C) Nest building behavior two days post-MPTP scored after 18 hours in the cage with a square cotton Nestlet. Saline-WT (n = 3), Saline-ASK1^−/−^ (n = 6), MPTP-WT (n = 3), MPTP-ASK1^−/−^ (n = 4). * p<0.05.

The motor manifestations of PD are due to the marked reduction in striatal dopamine content caused by the loss of dopaminergic nerve terminals in the striatum. Since ASK1 deficiency is associated with an attenuated response to MPTP toxicity, we investigated if ASK1^−/−^ mice have a better locomotor and behavioral performance following MPTP intoxication compared to wild-type littermates. First, performance on the rotarod was evaluated 1 day after saline or MPTP (20 mg/kg every 2 h×4) treatment. Compared to the poor performance of wild-type mice after MPTP, ASK1^−/−^ mice intoxicated with MPTP had longer latency to fall from the rotating bar indicating a better performance (Fig. 4A). Second, nest-building behavior, which requires orofacial and forelimb movements, is impaired after MPTP [20] and is dopamine-dependent [21], was evaluated 2 days after saline or MPTP (20 mg/kg every 2 h×4) administration. Compared with the 72% impairment in nest building ability in wild-type mice lesioned with MPTP vs saline, ASK1^−/−^ animals had significant preservation of this behavior after MPTP (Fig. 4B,C). These results are consistent with the biochemical and stereological indices of dopaminergic system preservation in MPTP lesioned ASK1^−/−^ mice.

Comparison of non-lesioned ASK1^−/−^ mice with wild-type littermates following saline administration showed a modest elevation of striatal dopamine content (17.2±0.62 vs 15.2±0.43 ng/mg, respectively; p<0.03) as described previously [22]. This was associated with a parallel increase in nigral dopamine neuron count (4984±347 vs 3215±276, respectively; p<0.05). Behaviorally, these animals are also reported to be hyperactive in a novel environment and to have superior performance on the rotarod [22], although their trend of better performance in this study was not significant (Fig. 4A, B).

### MPTP-induced glial activation is blunted in ASK1 null mice

**Figure 5:**
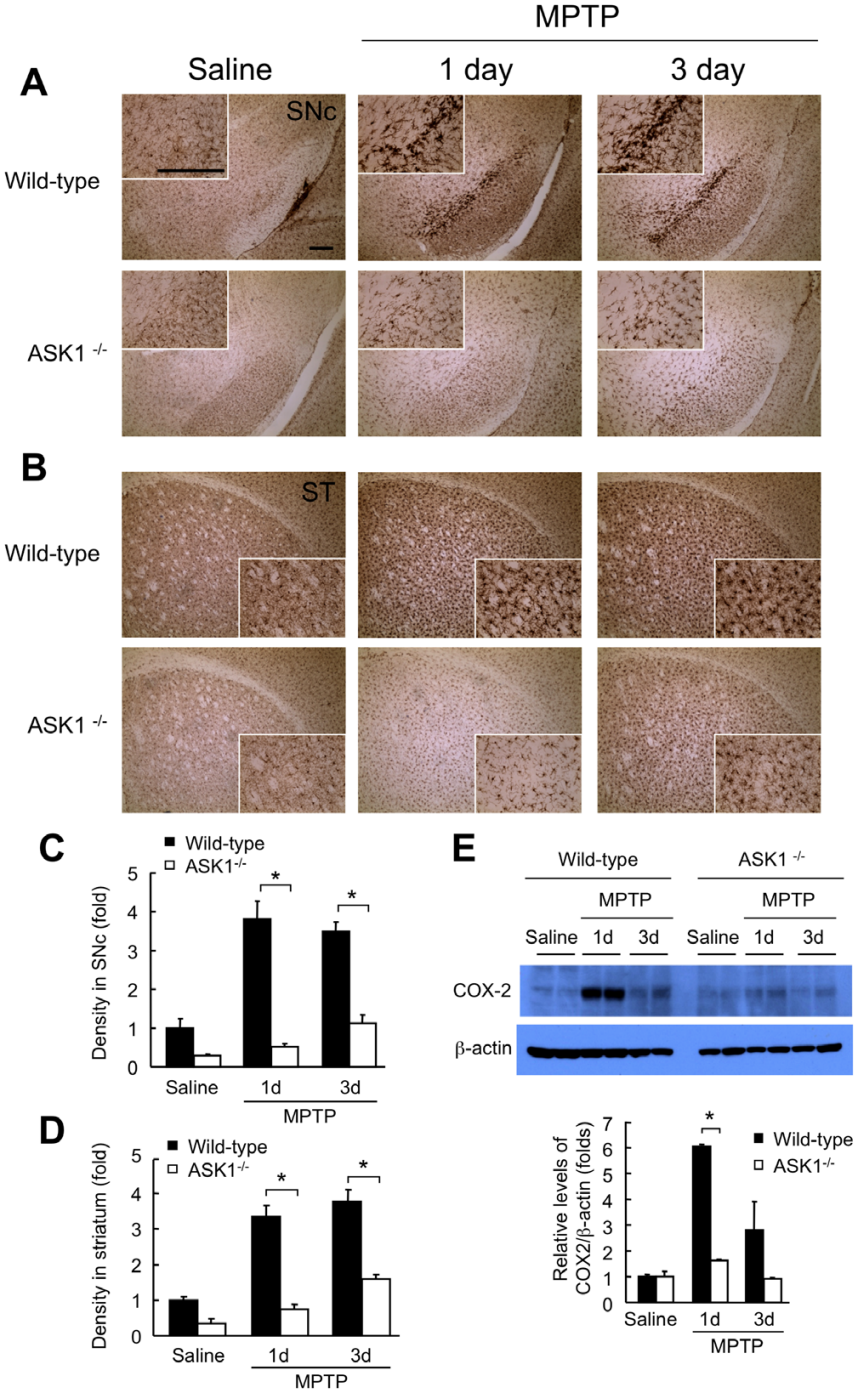
MPTP induced microglia activation is suppressed in ASK1^−/−^ mice. (A, B) Immunohistochemistry with the microglial marker Iba1 (polyclonal, 1∶2,000) of nigral (SNc) and striatal (ST) sections. Scale bar = 100 µm. (C) Quantification of nigral Iba1 immunopositivity. * p<0.001. (D) Quantification of striatal Iba1 immunopositivity. * p<0.001. Quantifications were done using ImageJ. (E) MPTP-induced COX-2 expression is attenuated in ASK1^−/−^ mice. Midbrains containing substantia nigra were homogenized and lysed for Western blotting for COX-2. Samples from two different mice per group are shown. * p<0.001. WT-Saline (n = 4), WT-MPTP-1 day (n = 4), WT-MPTP-3 day (n = 4); ASK1^−/−^-Saline (n = 3), ASK1^−/−^-MPTP-1 day (n = 4), ASK1^−/−^-MPTP-3 day (n = 3).

Microglia activation is associated with the pathogenesis of PD and is an important player in MPTP toxicity [23], [24]. To determine if ASK1 is involved in MPTP-induced microglia activation, wild-type and ASK1^−/−^ mice were subjected to the acute MPTP lesioning paradigm (20 mg/kg every 2 h×4), and brains were analyzed 1 day and 3 days after the last injection. Striatal and nigral sections were studied immunohistochemically using the microglial marker Iba1. Compared to the robust induction of Iba1 positive cells in the nigra of wild-type mice following MPTP, this response was significantly attenuated in ASK1^−/−^ mice (Fig. 5A, C). In the striatum, the activation of microglia after MPTP intoxication observed in wild-type mice was also markedly blunted in ASK1^−/−^ animals (Fig. 5B, D). Cyclooxygenase 2 (COX-2), the inducible form of the rate-limiting enzyme in prostaglandin synthesis, is increased in PD brains [25] and reportedly mediates microglia activation and subsequent dopamine neuron degeneration in the mouse MPTP model [26], [27]. Consistent with the significant attenuation in microglial activation following MPTP challenge in ASK1^−/−^, COX-2 expression detected by Western blotting with midbrain lysates showed minimal induction following MPTP administration in ASK1^−/−^ mice compared to the robust induction one day after MPTP in wild-type animals (Fig. 5E). The latter finding suggests that ASK1 is a key player in MPTP-induced COX-2 expression and subsequent microglia activation.

**Figure 6:**
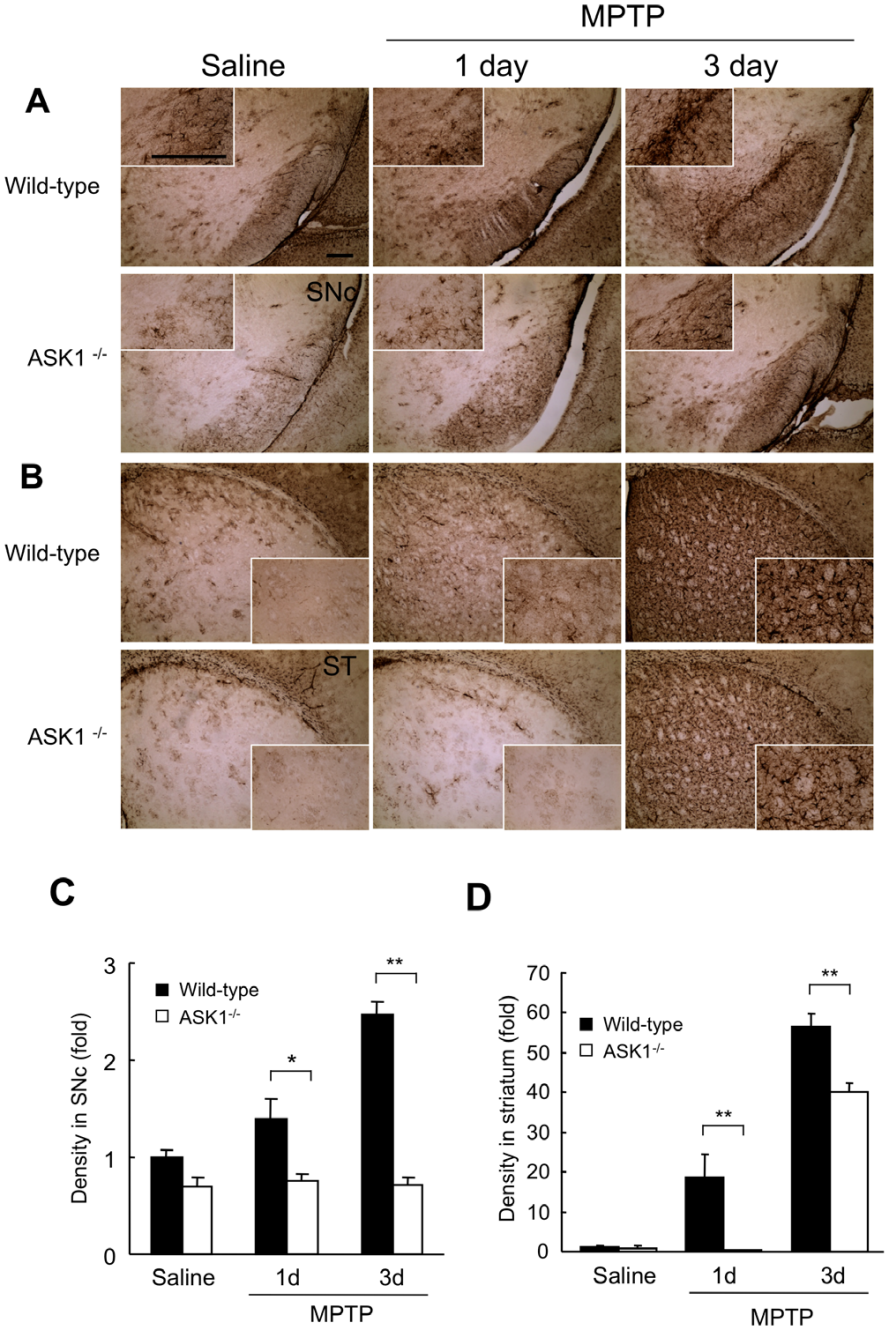
MPTP induced astrogilal activation is suppressed in ASK1^−/−^ mice. (A, B) Immunohistochemistry for GFAP in nigral (SNc) and striatal (ST) sections. Scale bar = 100 µm. (C) Quantification of nigral GFAP immunopositivity. * p<0.01; **<0.001. (D) Quantification of striatal GFAP immunopositivity. ** p<0.001. Quantifications were done using ImageJ. WT-Saline (n = 4), WT-MPTP-1 day (n = 4), WT-MPTP-3 day (n = 4); ASK1^−/−^-Saline (n = 3), ASK1^−/−^-MPTP-1 day (n = 4), ASK1^−/−^-MPTP-3 day (n = 3).

We also assessed activation of astroglial cells using immunohistochemistry for glial fibrillary acidic protein (GFAP) 1 day and 3 days after acute MPTP exposure. MPTP induced strong activation of GFAP positive cells in the nigra (Fig. 6A) and striatum (Fig. 6B) of wild-type mice particularly at 3 days, compared to a significantly blunted response in both these brain regions of ASK1^−/−^ mice, suggesting that ASK1 is required for MPTP-induced astrocyte activation as well.

## Discussion

In the present study, we have found that ASK1 deficiency partially protects the nigrostriatal dopaminergic system against MPTP-induced neurotoxicity and inhibits microglia and astrocyte activation. We also show that the behavioral benefits of ASK1 deletion correlate well with the observed cellular protection. These findings collectively suggest that ASK1 plays an important role in MPTP-induced toxicity in vivo.

Evidence is accumulating that chronic inflammation exacerbates the pathology in a number of neurodegenerative diseases including PD [28]. The innate immune cells of the brain, such as reactive astrocytes and microglia are abundant in the substantia nigra of PD [29], indicating a robust inflammatory state. Additionally, reactive microglial cells are seen in the basal ganglia in the 6-OHDA, MPTP and rotenone induced animal models of PD [30], [31], [32]. Further, α-synuclein, which aggregates in the characteristic inclusions in vulnerable neurons in PD patient brains, activates microglia and astrocytes in several PD models [33], [34], [35], [36]. However, it remains controversial whether activation of glial cells is favorable or detrimental to neurons. Our data suggest a detrimental role of activated glia in the neurodegenerative process, as the neuroprotection conferred by ASK1 deficiency correlates with a markedly attenuated glial response.

The mechanism by which MPTP leads to microglia activation remains obscure. Relevant to earlier reports that COX-2 mediates MPTP-induced microglia activation and subsequent dopamine neuron toxicity [26], [27], our results show that ASK1 is required for MPTP-induced COX-2 expression. This suggests that the role of ASK1 in regulating microglia activation is in part through regulating COX-2 expression [25]. Contrary to ASK1 deletion, JNK ablation reportedly does not interfere with microglia activation in the MPTP model, although targeted deletion of JNK protects dopaminergic neurons from MPTP toxicity [26]. Therefore, we speculate that the signal that leads to microglia activation is relayed from ASK1 to molecules other than JNK. In light of this notion, it is noteworthy that the phosphorylation of p38 MAP kinase, which is another kinase downstream of ASK1 [7], is observed within dopamine neurons in MPTP-lesioned mice, whereas JNK activation occurs primarily in microglia [37]. Further, the increased staining of phosphorylated p38 kinase in surviving nigral dopamine neurons in human brain sections from PD patients compared to age-matched controls provides further support for a role of p38 kinase in the degeneration of dopaminergic neurons [37]. A more direct relationship between ASK1 and p38 kinase has been shown in a study demonstrating that ASK1 is required for lipopolysaccharide (LPS)-induced activation of p38 but not of JNK in splenocytes and dendritic cells [38].

ASK1 is required for Toll-like receptor 4 (TLR4)-mediated mammalian innate immunity [38]. Splenocytes and dendritic cells isolated from ASK1^−/−^ mice lose the ability to express proinflammatory cytokines such as TNF-α, IL-1 and IL-6 following LPS stimulation. In addition, ASK1 is required for chemokine production in astrocytes through several TLRs, and ASK1 deficiency attenuates neuroinflammation in the experimental autoimmune encephalomyelitis model in mice [39]. Collectively, these results indicate that ASK1 is expressed in these immune/glial cells and plays a role in their cell signaling. Therefore, we postulate that the attenuated glial activation upon MPTP exposure in ASK1^−/−^ mice results from the effects of ASK1 deficiency in these cells. Based on Western blotting and RT-PCR with primary cell cultures, ASK1 is expressed in both microglia and astrocytes as well as in neurons including TH positive nigral dopaminergic neurons. We, therefore, hypothesize that ASK1 in dopamine neurons relays the signal(s) originated from MPP^+^ to produce the molecule(s) responsible for glial activation, although the nature of these molecules remains to be characterized. It is conceivable that α-synuclein released from dying dopamine neurons contributes to this event [33], [34]. In addition, ASK1 in glial cells may propagate the signal(s) conveyed from sick neurons finally resulting in glial activation and, in turn, further dopamine neuron injury.

In conclusion, our data provide evidence that ASK1 mediates microglia and astrocyte activation and MPTP-induced neurotoxicity, and that deletion of ASK1 prevents degeneration of DA neurons and glial activation. These findings suggest that ASK1 may serve as a potential target for the development of therapeutic interventions to slow or halt PD progression.

## Materials and Methods

### Cell and primary cultures

Human neuroblastoma SH-SY5Y cells (ATCC) were cultured in Dulbecco's modified Eagle's medium (DMEM) containing 10% fetal bovine serum (FBS) (Invitrogen) in a CO_2_ incubator at 37°C.

To prepare primary neuronal cultures, cortical tissue from E18 embryos of Sprague Dawley rats (Charles River) were recovered as described previously [40] under a protocol (# I08-034) approved by the Institutional Animal Care and Use Committee at the UMDNJ-Robert Wood Johnson Medical School. Tissues were minced and cells dissociated mechanically by gentle passaging through a flame-polished pasteur pipette in Neurobasal medium (Invitrogen). Cells were then passed through a strainer (45 µM pore size) and rinsed once in final culture medium (Neurobasal medium with B27 serum-free supplements, 0.5 mM L-glutamine, 0.1 IU/ml penicillin and 10 µg/ml streptomycin). Dissociated cells were plated at a density of 2×10^5^ per cm^2^ in 100 µg/ml poly-D-lysine coated plates, and 1 µM cytosine arabinoside was added to the medium.

### *In vitro* ASK1 Activity Assay

Cells were challenged with 5 mM MPP^+^ (Sigma) for the indicated times, and lysates were processed for immunoprecipitation with anti-ASK1 antibody (H-300, Santa Cruz Biotechnology). The immunoprecipitated complex was used for in vitro kinase assay in a reaction buffer consisting of 20 mM Tris-HCl (pH 7.5), 20 mM MgCl_2_, 5 µCi [γ-^32^P] ATP for 20 min at 30°C using myelin basic protein (MBP) (40 µg/ml) as substrate. Samples were resolved in SDS-PAGE and subjected to autoradiography.

### Animals and MPTP Administration

ASK1 knockout mice were maintained on the C57BL/6J background as described previously [7]. All animals were handled in accordance with the NIH guidelines for the use of laboratory animals under a protocol (# I07-011-2) approved by the Institutional Animal Care and Use Committee at the UMDNJ-Robert Wood Johnson Medical School. Three-month-old wild-type and ASK1^−/−^ male mice were challenged intraperitoneally with MPTP dissolved in sterile saline. Control animals received saline injections. Injection paradigms and doses are described in the Results section.

### Western Blot

Cells and tissues were lysed with RIPA buffer (50 mM Tris, pH 8.0, 150 mM NaCl, 1% NP-40, 0.1% SDS, 0.5% sodium deoxycholate) containing phosphatase inhibitor cocktail set II (Calbiochem, La Jolla, CA) and protease inhibitor cocktail set V (Calbiochem, La Jolla, CA). Lysates were separated in 4–12% gradient gel (Invitrogen). Primary antibodies used for Western blots and immunohistochemistry in these studies were as follows: anti-ASK1 (H-300) (Santa Cruz Biotechnology), anti-phospho-ASK1 (generated against the ASK1 epitope that contains p-Thr845) [41], anti-TH (Sigma), anti-GFAP (DAKO), anti-Iba1 (Wako), anti-COX-2 (BD Bioscience), and anti-β-actin (Sigma). Secondary antibodies (Sigma), anti-rabbit IgG-HRP or anti-mouse IgG-HRP were used with an enhanced chemiluminescence (ECL) kit (PerkinElmer LAS) for signal generation on X-ray film.

### Immunohistochemistry and Stereology

Mice were perfused transcardially with PBS, and brains were removed and post-fixed in 4% paraformaldehyde at 4°C overnight. Free-floating, 40 µm thick coronal sections were incubated with the indicated primary antibodies overnight, followed by staining with the ABC kit (Vector Laboratories).

For counting nigral dopaminergic neurons, coronal 40-µm-thick sections were cut through the entire substantia nigra (SN). The SN pars compacta and medial ventral tegmental area regions were outlined for neuron counting according to previous anatomical demarcations of the midbrain dopaminergic neurons in the mouse [42]. Every fourth section through the rostral–caudal extent of the SN was stained with an antibody against tyrosine hydroxylase (TH, 1∶4000; Sigma) and processed with the ABC method. Tissue sections were counterstained with cresyl violet, a Nissl stain, and cover-slipped. StereoInvestigator software (version 8.0. MicroBrightfield Inc., Williston, VT) was used to count TH-immunoreactive (TH-IR) cells and Nissl-stained cells. Cells were counted with a 100× oil immersion objective (1.3 NA) using a Leica DMRE microscope. The cell counting frame was 50×50×5 µm with a 1 µm upper and lower guard zone. A cell was defined as a TH-IR soma with a clearly visible unstained nucleus. For Nissl-stained cells, a cell was defined as a non-TH-containing soma in focus within the counting frame. The TH cell counts were taken from 6 sections, spaced 4 apart (120 µm) and 200–250 cells were counted in the SN on one side of the brain. For Nissl cell counting, the same sections were examined. TH-containing cells represent dopaminergic neurons, and cresyl-violet stained neurons represent all other nondopaminergic neurons.

### Measurement of Striatal Dopamine, MPP^+^ and TH

Mice were killed after MPTP or saline injections at time points specified in the Results section, and their striata were quickly dissected. Striatal dopamine and its metabolites were measured by HPLC-electrochemical detection as described previously [43]. Striatal levels of MPP^+^ were measured by HPLC using UV detection. Striatal tyrosine hydroxylase content was measured by an enzyme-linked immunosorbent assay method [44].

### Behavior Assessments

Motor coordination and motor learning were measured by the rotarod test. Mice were placed on a rotating cylinder (diameter = 4.5 cm) with a coarse surface for a firm grip and tested for three trials with an accelerating speed of 0.2 rpm/second, increasing from 4 to 40 rpm. A cut-off time of 3 min and an inter-trial interval of 60 min were used. The latency of time spent on the rod before falling was measured.

To assess nest-building performance, each mouse was housed in a cage containing a single block of nesting material (NestletsTM, Ancare Corp) for 18 h. Each cage was then scored blindly depending on the condition of nesting material on a scale ranging from 0 = non shredded to 5 = maximally shredded [45].

### Statistical Analysis

Two-sample comparisons were carried out using Student's *t*-test and multiple comparisons were done using one-way ANOVA followed by the Newman-Keuls multiple range test. Data are presented as means ± SEM and statistically significant differences were accepted at the p<0.05 level.
